# Environmental Exposure to Waterborne Pollutants and Colorectal Cancer Risk in Lebanon

**DOI:** 10.3390/toxics13090792

**Published:** 2025-09-17

**Authors:** Magie Tamraz, Najib Al Ghossaini, Sally Temraz

**Affiliations:** 1 Department of Epidemiology and Population Health, Faculty of Health Sciences, American University of Beirut, Riad El Solh, Beirut 1107, Lebanon; 2 Department of Internal Medicine, Ain Wazein Medical Village, Chouf P.O. Box 1503-2010/02, Lebanon; najibaal@hotmail.com; 3 Division of Oncology/Hematology, Department of Internal Medicine, American University of Beirut Medical Center, Riad El Solh, Beirut 1107, Lebanon

**Keywords:** arsenic, colorectal cancer, contaminants, *E. coli*, mercury, nitrates, tetrahalomethanes, water

## Abstract

Colorectal cancer (CRC) has a complex etiology involving genetic, lifestyle, and environmental factors. This study investigates the association between exposure to water contaminants and the risk of developing CRC in Lebanon. Population Attributable Fraction (PAF) was calculated using exposure prevalence from environmental reports and water quality assessments. Relative risks (RR) were obtained from multiple meta-analyses and epidemiological studies to quantify the contribution of five water contaminants to CRC incidence: nitrates, mercury, arsenic, trihalomethanes (THMs), and microbial pollutants (*E. coli*). A synthetic cohort was simulated using national exposure data and probabilistic techniques, employing multivariate logistic regression models to ensure precise CRC risk and controlling for lifestyle covariates. Adjusted PAF values were calculated using regression data to determine the proportion of CRC cases attributable to each water contaminant. THMs and *E. coli* showed the strongest association with CRC, with adjusted PAF values of 25.76% and 23.65%, respectively. Exposure to nitrates, arsenic, and mercury contributed less to CRC risk (1.02%, 0.52% and 2.20%, respectively). Multivariate regression confirmed that THMs and *E. coli* remained the strongest independent predictors of CRC risk (OR = 1.37, *p* = 0.001) and (OR = 1.79, *p* < 0.0001) among water contaminants, respectively. Our model-based approach carries limitations due to reliance on indirect exposure and risk estimates, which may introduce uncertainty stemming from data gaps and underlying assumptions. This study highlights the importance of water quality management in CRC prevention as exposure to drinking water contaminants contributes meaningfully to disease burden.

## 1. Introduction

Colorectal cancer (CRC) ranks third in global cancer incidence and is the second leading cause of cancer-related mortality, accounting for 9.4% of cancer-related deaths worldwide [1]. Despite significant progress in cancer chemotherapy and the development of new delivery systems for antitumor drugs, success in the treatment of CRC remains modest [2,3]. CRC can be classified into three types: sporadic, hereditary, and colitis-associated. Its development is influenced by both modifiable and non-modifiable risk factors. Age, sex, race, and family history, including conditions such as familial adenomatous polyposis and Lynch syndrome, are well-established non-modifiable contributors [4]. Modifiable factors include lifestyle behaviors such as smoking, alcohol consumption, physical inactivity, obesity, and diets high in red and processed meats [4].

Environmental exposures, particularly water contamination, have emerged as a growing concern. In Lebanon, deteriorating infrastructure and untreated wastewater have compromised public water sources, prompting reliance on private wells and bottled water [5,6]. The presence of heavy metals, nitrates, and disinfection by-products such as trihalomethanes (THMs) in contaminated water have been associated with increased CRC risk in international studies [7,8,9]. Also, bacterial contaminants such as *Escherichia coli*, *Pseudomonas aeruginosa*, and fecal coliforms may contribute to chronic inflammation, a known driver of CRC progression [10].

Between 2005 and 2016, Lebanon experienced a notable rise in age-standardized CRC rates; from 16.3 to 23.2 per 100,000 in males, and from 13.0 to 20.2 per 100,000 in females, reflecting an annual increase of approximately 4.4% [11]. Despite this trend, the role of environmental exposures in CRC etiology remains poorly understood. Most prior research has focused on bladder cancer, leaving the link between water-borne contaminants and CRC largely unexplored. This study aims to investigates the potential association between water contamination and CRC risk in the Lebanese population, addressing a critical gap in regional cancer epidemiology.

## 2. Materials and Methods

### 2.1. Study Design

This retrospective modeling study aimed to quantify the contribution of environmental exposures to CRC incidence in Lebanon. We focused on five waterborne contaminants: THMs, nitrates, heavy metals (arsenic, mercury), and microbial contaminants based on prior evidence linking them to CRC development. The Population Attributable Fraction (PAF) was calculated for each contaminant to estimate its contribution to disease burden.

To account for age-related differences in CRC susceptibility, we stratified the synthetic population into three groups:

- ≤40 years (younger adults);
- 41–60 years (middle-aged adults);
- ≥61 years (older adults).

This stratification follows the framework outlined in Bray et al. which provides global cancer incidence and mortality estimates across different age groups [12].

### 2.2. Exposure Assessment

Exposure data were sourced from environmental reports and water quality assessments conducted in Lebanon. These datasets are national in scope, though some are regionally focused (e.g., Beirut and Mount Lebanon). Table 1 summarized the sources used. Contaminant levels were categorized according to United States Environmental Protection Agency (USEPA) Maximum Contaminant Level (MCL):

- Arsenic: 10 ppb;
- Nitrates: 10 mg/L;
- THMs: 80 ppb;
- Mercury: 2 ppb;
- *E. coli*: 0 colonies/100 mL.

Some datasets, such as THMs from 2007 and mercury from 2017, may appear outdated. However, to our knowledge, no more recent national-level data are publicly available. This limitation is explicitly addressed in the manuscript, and we emphasize the urgent need for updated environmental surveillance in Lebanon. Geographic variability was partially accounted for by incorporating data from multiple regions, though we recognize that generalizability remains limited.

### 2.3. Synthetic Cohort Generation and Risk Modeling

Due to the absence of accessible individual-level health data in Lebanon, we constructed a synthetic cohort of 10,000 individuals using probabilistic simulation techniques in R (https://posit.cloud, version 4.3.3 (2024-02-29)). To support the development of the simulation model, we utilized Microsoft Copilot (version 1.25064.139.0, accessed via Windows 11) to generate the initial R code structure and logic. The model was subsequently implemented and executed in R, where we conducted output verification and performed sensitivity analyses to assess the robustness of the simulation outcomes.

Each individual was assigned binary exposure variables for environmental and lifestyle risk factors using binomial distributions informed by national prevalence rates and concentration ranges reported in governmental and peer-reviewed sources. The assigned prevalence values were as follows:

- Arsenic: 5% [13];
- Mercury: 23% [14];
- Nitrates: 15% [13];
- THMs: 94.7% [15];
- *E. coli*: 39% [14];
- Smoking: 35% [16];
- Processed meat intake: 74.5% [17];
- Physical inactivity: 64% [18].

Each individual’s probability of developing CRC was modeled using a logistic function based on weighted log-odds coefficients derived from published literature. The outcome variable (CRC status) was simulated using the inverse logit of the combined risk score. Covariate relationships were modeled using a dependency matrix and Cholesky decomposition to reflect real-world correlations between exposures and lifestyle factors.

Relative risk (RR) estimates for each contaminant were sourced from peer-reviewed meta-analyses and epidemiological studies:

- Arsenic: Tsuji et al. [19];
- Nitrates: Assaf et al. [20];
- Mercury: Obeid et al. [21];
- THMs: Kumari et al. [22];
- *E. coli*: Global review [23].

RR ranges were converted to midpoint values for each age group (≤40, 41–60, ≥61 years), and unadjusted Population Attributable Fractions (PAFs) were calculated using the standard formula:

PAF = Pe × (RR − 1)/[Pe × (RR − 1) + 1],(1)

where

- Pe represents the prevalence of exposure in the population;
- RR is the relative risk of CRC associated with each contaminant.

To derive age-specific RR values from overall RR estimates, we applied a proportional scaling method. This approach adjusts RR values across age strata based on known patterns of CRC incidence, allowing us to estimate stratified PAFs even in the absence of age-specific exposure data. The scaling was informed by global CRC incidence curves and validated against age-specific cancer risk distributions reported in Bray et al. [12].

Although our model assumes independent effects of each contaminant, we acknowledge that real-world exposures may involve synergistic or antagonistic interactions. These complex dynamics have been documented in the recent literature, including Lagunas-Rangel et al., who describe how pollutant mixtures can interact through shared biological pathways to influence cancer development [24].

### 2.4. Statistical Analysis, Adjusted PAF and Sensitivity Testing

Multivariate logistic regression was performed to estimate adjusted odds ratios (ORs), 95% confidence intervals (CIs), and *p*-values for each exposure variable. The model included both environmental contaminants and lifestyle factors previously identified as contributing to CRC risk. All predictors were entered simultaneously to account for confounding effects and to isolate the contribution of each variable.

Adjusted PAFs were calculated using the same formula as above, substituting the regression-derived ORs for RR values. This allowed us to estimate the proportion of CRC cases attributable to each contaminant while controlling for lifestyle risk factors. The adjusted PAFs reflect the net contribution of each exposure in a multivariate context, providing a more realistic estimate of disease burden.

To evaluate the robustness of our PAF estimates, we conducted sensitivity analyses by varying both exposure prevalence and relative risk (RR) values within plausible bounds derived from the literature. Specifically, RR values were adjusted ±10% from their midpoint estimates, and prevalence rates were varied within documented national ranges. For each contaminant, PAFs were recalculated under these alternative scenarios. This approach allowed us to assess the stability of our findings and identify which exposures exert the greatest influence on CRC burden under uncertainty. Full sensitivity results and code are provided in the Supplementary Materials.

The full R code used for simulation, regression modeling, and PAF calculations is provided in the Supplementary Materials to ensure transparency and reproducibility.

## 3. Results

### 3.1. Water Contaminant Exposure in the Lebanese Population

Recent national water quality assessments reveal widespread exposure to several contaminants in Lebanon. Mercury concentrations exceeded WHO limits in 23% of sampled sites. According to the WHO/UNICEF survey, 15% of the Lebanese population is exposed to nitrates and 5% to arsenic in drinking water [13]. A regional study conducted in Beirut and Mount Lebanon revealed microbial contamination in over 60% of samples, with *E. coli* detected in 39% of treated water samples [14]. Mercury concentrations exceeded WHO limits in 23% of sampled sites [14]. Additionally, Semerjian et al. found that 94.7% of water networks across Lebanon exceed the USEPA’s concern threshold for THM contamination [15] (Table 2).

### 3.2. Age-Stratified Risk Estimates for Colorectal Cancer

RR estimates for CRC were stratified by age using a proportional scaling method informed by global incidence trends. Table 2 presents the estimated RR ranges across three age groups: ≤40, 41–60, and ≥61 years. The highest CRC risk was observed in older adults, particularly for THM exposure (RR: 1.26–1.43) and *E. coli* contamination (RR: 1.65–2.20).

### 3.3. Adjusted Multivariate Regression Model

To isolate the independent contribution of water contaminants to CRC risk, a multivariate logistic regression model was constructed, adjusting for lifestyle factors including smoking, processed meat consumption, and physical inactivity. Table 3 summarizes the odds ratios (ORs), confidence intervals, and *p*-values.

Significant associations were observed for THMs, *E. coli*, smoking, processed meat intake, and inactivity. THMs were associated with a 36.6% increase in CRC odds, while *E. coli* exposure nearly doubled the risk. Arsenic and nitrate showed elevated ORs but did not reach statistical significance. Mercury had no meaningful association.

**Figure 1:**
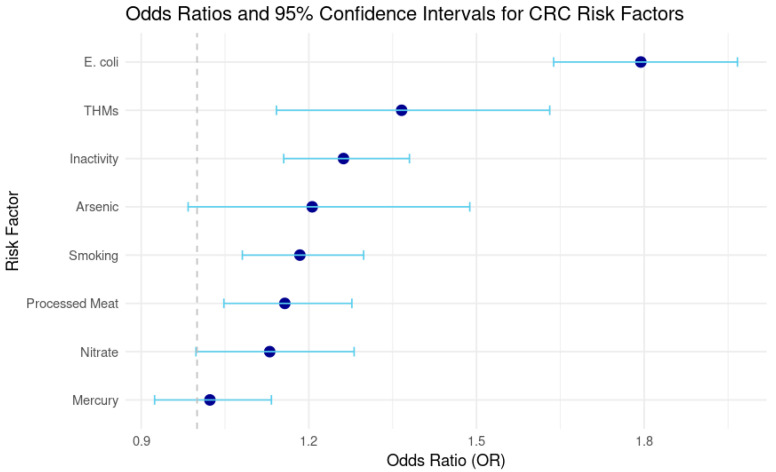
Odds Ratios and 95% Confidence Intervals for Colorectal Cancer Risk Factors. Navy blue dots show odds ration and blue lines show 95% confidence intervals.

Figure 1 presents a forest plot of odds ratios and 95% confidence intervals, illustrating the strength and precision of associations across all modeled risk factors.

### 3.4. Adjusted Population Attributable Factor (PAF) Estimates

PAF estimates were calculated to quantify the proportion of CRC cases attributable to each contaminant, both unadjusted and adjusted for lifestyle confounders. Table 4 presents these values across age groups and overall.

**Figure 2:**
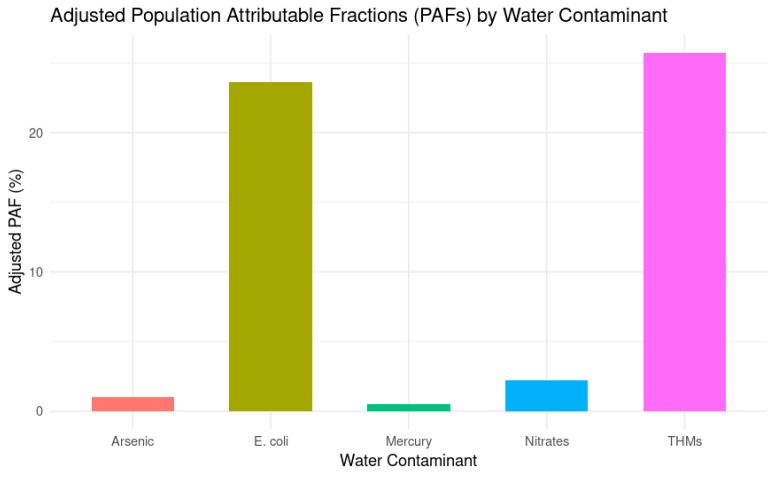
Adjusted Population Attributable Fractions (PAFs) by Water Contaminant.

Figure 2 provides a visual comparison of adjusted PAFs across contaminants, highlighting THMs and *E. coli* as the most impactful exposures

Adjusted PAFs revealed that *E. coli* and THMs were the most impactful contaminants, accounting for 23.65% and 25.76% of CRC cases, respectively. These findings suggest that nearly one in four CRC cases could be attributed to each of these exposures independently. *E. coli* showed a clear age-related increase, with unadjusted PAFs rising from 20.47% in individuals aged ≤40 to 26.51% in those aged ≥61. THMs followed a similar pattern, with unadjusted PAFs increasing from 13.16% to 24.63% across age groups. After adjusting for lifestyle confounders, the PAF for THMs rose substantially, indicating that its true impact may have been previously masked by overlapping risk factors.

In contrast, mercury showed modest unadjusted PAFs that dropped sharply after adjustment, from 2.25% to just 0.52%, suggesting that its apparent contribution may be influenced by other exposures or behaviors. Arsenic remained low across all age groups, peaking at 0.74% in older adults, while its adjusted PAF increased slightly to 1.02%, indicating a limited but independent role. Nitrates displayed consistent PAF values across models, with an adjusted PAF matching the overall unadjusted estimate of 2.20%, pointing to a stable and modest association with CRC risk.

To assess the robustness of our PAF estimates, we conducted a sensitivity analysis by varying relative risk values ±10% for each contaminant. The results confirmed that *E. coli* and THMs remained the most influential contributors to CRC burden across all tested scenarios. Mercury showed high uncertainty, with a negative PAF under conservative assumptions, while arsenic and nitrates demonstrated stable but modest effects. Full results and code are provided in the Supplementary Materials.

## 4. Discussion

The results of this study provide the first comprehensive assessment of water contaminants’ impact on CRC risk in the Lebanese population. The findings reveal that while heavy metals (arsenic and mercury) and nitrates contribute mildly to CRC incidence, with adjusted PAF values of 1.02% for arsenic, 0.52% for mercury, and 2.20% for nitrates, THMs and microbial contaminants present a moderate impact, with adjusted PAF values of 25.76% and 23.65%, respectively. Thus, the worsening water contamination crisis in Lebanon could be playing a significant role in the increasing incidence of CRC, emphasizing the need for immediate public health interventions.

Several epidemiological studies have highlighted the associations between water pollutants and CRC risk. For instance, Essien et al. assessed the impact of nitrate ingestion and found an OR of 1.11 (95% CI: 1.04, 1.17) for CRC risk [25]. Similarly, Kasmi et al. reported an arsenic-related CRC risk with an OR of 1.49 (95% CI: 1.20–1.83) in men and 1.42 (95% CI: 1.13–1.76) in women [26]. Helte et al.’s recent systematic review found THM exposure to be significantly associated with CRC risk, with a RR of 1.15 (95% CI: 1.07, 1.24) [27]. Finally, Bonnet et al. determined that *E. coli* contamination increases CRC risk, reporting an OR of 2.5 (95% CI: 1.5, 4.2) [28]. While these studies validate the carcinogenic potential of water contaminants, their findings were derived from populations outside Lebanon, with varying environmental and genetic backgrounds. This study’s PAF calculations integrate both relative risk estimates and exposure prevalence, ensuring that the impact of contaminants is quantified in the Lebanese population.

To enhance the accuracy of the attributable risk estimates, this study employed multivariate regression analyses that adjusted for key lifestyle factors, including smoking prevalence, dietary patterns, and physical inactivity. The adjusted findings reaffirm that THMs are a significant predictor of CRC risk (OR = 1.37, *p* = 0.001), even after accounting for potential confounding lifestyle variables. Exposure to *E. coli* also remains highly significant (OR = 1.79, *p* < 0.0001), underscoring persistent concerns regarding microbial contamination in drinking water. These results indicate that although lifestyle factors play a role in CRC development, the independent carcinogenic impact of THMs and microbial contaminants is considerable.

The adjusted PAF values for mercury and arsenic differed notably from their unadjusted counterparts. Mercury’s adjusted PAF dropped from 2.25% to 0.52%, suggesting that its apparent contribution may be confounded by other exposures or behaviors. In contrast, arsenic’s adjusted PAF increased slightly despite low unadjusted values, indicating a limited but independent role. These shifts underscore the importance of controlling for lifestyle factors when estimating attributable risk.

Water contamination in Lebanon is driven by an aging infrastructure, agricultural runoff, industrial pollution, and insufficient regulatory enforcements. An aging infrastructure has resulted in many of Lebanon’s water supply systems being old and poorly maintained, with cracks and leaks in pipes that can allow contaminants to enter the water supply. Pollutants from various sources, including agricultural runoff, sewage discharge, and industrial waste, contaminate water bodies. Insufficient monitoring and regulation of water quality often resulted in undetected contamination, with irregular testing and weak enforcement of water standards. Economic and political instability has further hindered efforts to maintain and upgrade the infrastructure. These factors highlight the need for improved water treatment infrastructure, stricter regulations, and regular monitoring to ensure the safety and quality of drinking water in Lebanon. In addition, public awareness campaigns are needed to educate communities about the risks associated with contaminated drinking water and promote safe water practices.

This study is subject to several methodological and contextual limitations. First, the PAF for each drinking water contaminant was calculated under the assumption of independent contributions to CRC risk. However, interactions among contaminants may influence risk in more complex and interdependent ways. Future studies should explore potential synergistic or antagonistic effects among contaminants to better understand cumulative exposure risks. Furthermore, the analysis was based on a simulation model which, although useful for exploring exposure scenarios, depends on assumptions that may constrain its accuracy in real-world settings. These include uncertainties in synthetic data generation, reliance on external RR estimates, and lack of differentiation between short- and long-term exposures. Second, exposure data were estimated and may not accurately represent current conditions across Lebanon, particularly in light of geographic variability and reliance on outdated sources such as Semerjian et al. (2007) [15]. Additionally, the RR values were derived from studies that may not reflect the demographic and lifestyle characteristics of the Lebanese population, and the analysis did not differentiate between short-term and long-term exposures. These limitations may affect the precision and generalizability of the study’s findings. Therefore, the associations presented should be interpreted with caution, and not as definitive causal relationships.

## 5. Conclusions

Water contamination remains a significant public health concern in Lebanon, and our study underscores its potential impact on CRC risk. By assessing the contribution of various contaminants, including THMs, microbial pollutants, arsenic, nitrates, and mercury, we highlight the urgent need for improved water quality management and regulatory enforcement. The findings suggest that water pollutants may play a substantial role in CRC incidence, reinforcing the need for targeted interventions in water treatment infrastructure and environmental policies.

To translate these findings into actionable steps, we recommend implementing a national water quality reporting system, increasing the frequency of contaminant monitoring, particularly in high-risk regions, and upgrading aging water infrastructure. Public awareness campaigns should also be launched to educate communities about the risks of contaminated drinking water and promote safe water practices.

Considering the intricate relationship between environmental contaminants and lifestyle factors, future research in Lebanon should focus on improving exposure assessment methods by integrating longitudinal data and biomonitoring techniques. Such efforts will enhance the precision of risk estimates and help identify vulnerable populations. Achieving a more accurate understanding of the risks associated with specific contaminants will be critical for developing effective cancer prevention strategies and safeguarding the quality of drinking water for the population.

## Figures and Tables

**Table 1:** Summary of Water Contaminant Exposure Studies Used in Risk Assessment.

Contaminant	Reference	Study Year	Sample Size	Study Location
Nitrates	WHO/UNICEF [13]	2016	Drinking water samples	Nationwide
Arsenic	WHO/UNICEF [13]	2016	Drinking water samples	Nationwide
Mercury	Abou Abbass et al. [14]	2017	79 drinking water samples	Beirut & Mount Lebanon
THMs	Semerjian et al. [15]	2007	50 water treatment plants	Nationwide
*E. coli*	Abou Abbass et al. [14]	2021	79 drinking water samples	Beirut & Mount Lebanon

**Table 2:** Population at Risk and Relative Risk (RR) for Colorectal Cancer Based on Water Contaminant Exposure and Age Groups.

Contaminant	Population at Risk (%)	RR ≤40	RR 41–60	RR ≥61	Overall RR
Arsenic	5%	0.97–1.02	1.02–1.07	1.12–1.18	1.02–1.07
Mercury	23%	1.00–1.09	1.05–1.15	1.16–1.26	1.05–1.15
Nitrates	15%	1.04–1.14	1.10–1.20	1.21–1.32	1.10–1.20
THMs	94.7%	1.09–1.23	1.15–1.31	1.26–1.43	1.15–1.30
*E. coli*	39%	1.42–1.90	1.50–2.00	1.65–2.20	1.5–2.0

**Table 3:** Multivariate Logistic Regression Results.

Variable	OR	95% CI	*p*-Value
Arsenic	1.206	0.984–1.488	0.075
Nitrate	1.130	0.998–1.281	0.055
THMs	1.366	1.142–1.631	0.001
Mercury	1.023	0.924–1.133	0.665
*E. coli*	1.794	1.638–1.967	<0.0001
Smoking	1.184	1.081–1.298	0.0003
Processed Meat	1.157	1.048–1.277	0.004
Inactivity	1.262	1.155–1.380	<0.0001

**Table 4:** Adjusted and unadjusted PAF Estimates for Water Contaminants.

Contaminant	PAF ≤40 (%)	PAF 41–60 (%)	PAF ≥61 (%)	PAF Overall (%)	Adjusted Overall (%)
Arsenic	−0.03	0.22	0.74	0.22	1.02
*E. coli*	20.47	22.63	26.51	22.63	23.65
Mercury	1.02	2.25	4.61	2.25	0.52
Nitrates	1.33	2.20	4.58	2.20	2.20
THMs	13.16	17.89	24.63	17.56	25.76

## Data Availability

The original contributions presented in this study are included in the article. Further inquiries can be directed to the corresponding author.

## Supplementary Materials

The following supporting information can be downloaded at: https://www.mdpi.com/article/10.3390/toxics13090792/s1, Supplementary File S1.

## Abbreviations

The following abbreviations are used in this manuscript:

CI	Confidence interval
CRC	Colorectal Cancer
IARC	International Agency for Research on Cancer
MCL	Maximum contaminant level
OR	Odds ratio
PAF	Population attributable fraction
RR	Relative risk
THM	Trihalomethanes
USEPA	United States Environmental Protection Agency
