# Supplementary material for: Environmental Exposure to Waterborne Pollutants and Colorectal Cancer Risk in Lebanon
Source: Toxics. 2025 Sep 17;13(9):792. doi: 10.3390/toxics13090792 (PMC12474167; doi:10.3390/toxics13090792)
Supplement: Supplementary file 1 [file toxics-13-00792-s001.zip › toxics-3842240-supplementary.pdf]

This document provides detailed information on the simulation model, exposure assignment, age-specific scaling, PAF calculations, and sensitivity analysis used in our study on colorectal cancer (CRC) risk in Lebanon. All code was written in R (version 4.3.1) and is included below for full reproducibility.

1. Synthetic Cohort Generation

We simulated a cohort of 10,000 individuals using binomial distributions to assign binary exposure status for environmental and lifestyle risk factors. Prevalence rates were derived from national water quality assessments and health surveys.

Assigned Prevalence Values

Variable	Prevalence (%)
Arsenic	5
Mercury	23
Nitrates	15
THMs	94.7
E. coli	39
Smoking	35
Processed Meat	74.5
Physical Inactivity	64

Simulation Code

```
set.seed(42)
n <- 10000

arsenic <- rbinom(n, 1, 0.05)
nitrate <- rbinom(n, 1, 0.15)
thms <- rbinom(n, 1, 0.947)
mercury <- rbinom(n, 1, 0.23)
ecoli <- rbinom(n, 1, 0.39)
smoking <- rbinom(n, 1, 0.35)
processed_meat <- rbinom(n, 1, 0.745)
inactivity <- rbinom(n, 1, 0.64)

log_odds <- (
  0.044 * arsenic +
  0.140 * nitrate +
  0.281 * thms +
```

```
  0.095 * mercury +
  0.560 * ecoli +
  0.131 * smoking +
  0.199 * processed_meat +
  0.223 * inactivity
)

p <- 1 / (1 + exp(-log_odds))
outcome <- rbinom(n, 1, p)

df <- data.frame(
  Outcome = outcome,
  Arsenic = arsenic,
  Nitrate = nitrate,
  THMs = thms,
  Mercury = mercury,
  Ecoli = ecoli,
  Smoking = smoking,
  ProcessedMeat = processed_meat,
  Inactivity = inactivity
)
```

2. Multivariate Logistic Regression

We fit a logistic regression model to estimate adjusted odds ratios (ORs) for each variable.

```
library(broom)
model <- glm(Outcome ~ ., data = df, family = binomial)

result <- tidy(model, exponentiate = TRUE, conf.int = TRUE) %>%
  filter(term != "(Intercept)") %>%
  rename(
    Variable = term,
    `Odds Ratio (OR)` = estimate,
    `Lower CI` = conf.low,
    `Upper CI` = conf.high,
    `p-value` = p.value
  )
print(result)
```

3. Age-Stratified RR Scaling

We scaled relative risk (RR) ranges across age groups using multipliers derived from global CRC incidence curves (Bray et al.).

```
contaminants <- c("Arsenic", "Mercury", "Nitrates", "THMs", "E. coli")
rr_low <- c(1.02, 1.05, 1.10, 1.15, 1.5)
rr_high <- c(1.07, 1.15, 1.20, 1.30, 2.0)

age_multipliers <- list(
  young = 0.95,
  middle = 1.00,
  older = 1.10
)

scale_rr <- function(rr_lo, rr_hi, multiplier) {
  low <- round(rr_lo * multiplier, 2)
  high <- round(rr_hi * multiplier, 2)
  return(paste0(low, "–", high))
}

rr_young <- mapply(scale_rr, rr_low, rr_high, MoreArgs = list(multiplier = age_multipliers$young))
rr_middle <- mapply(scale_rr, rr_low, rr_high, MoreArgs = list(multiplier = age_multipliers$middle))
rr_older <- mapply(scale_rr, rr_low, rr_high, MoreArgs = list(multiplier = age_multipliers$older))
rr_overall <- paste0(rr_low, "–", rr_high)

rr_table <- data.frame(
  Contaminant = contaminants,
  RR_Overall = rr_overall,
  RR_LessThan40 = rr_young,
  RR_41_60 = rr_middle,
  RR_Over61 = rr_older
)
print(rr_table)
```

4. PAF Calculations

Unadjusted and adjusted PAFs were calculated using the standard formula:

$$ \text{PAF} = \frac{Pe \times (RR - 1)}{Pe \times (RR - 1) + 1} $$

```
compute_paf <- function(RR, prevalence) {
  (prevalence * (RR - 1)) / ((prevalence * (RR - 1)) + 1)
}
```

Adjusted PAFs used ORs from the regression model in place of RR values.

5. Sensitivity Analysis Procedure

To evaluate the robustness of our PAF estimates, we varied each adjusted OR ±10% and recalculated PAFs. This allowed us to assess how sensitive each contaminant's contribution to CRC burden was to uncertainty in risk estimates.

```
sensitivity_rr <- function(rr, prevalence) {
  rr_low <- rr * 0.90
  rr_high <- rr * 1.10
  paf_low <- compute_paf(rr_low, prevalence)
  paf_high <- compute_paf(rr_high, prevalence)
  return(data.frame(RR_Low = rr_low, RR_High = rr_high,
                    PAF_Low = round(100 * paf_low, 2),
                    PAF_High = round(100 * paf_high, 2)))
}

adjusted <- data.frame(
  Contaminant = c("Arsenic", "Nitrate", "THMs", "Mercury", "E.coli"),
  OR = c(1.206305, 1.129867, 1.366487, 1.022842, 1.794397),
  Prevalence = c(0.05, 0.15, 0.947, 0.23, 0.39)
)

sensitivity_results <- lapply(1:nrow(adjusted), function(i) {
  row <- adjusted[i, ]
  result <- sensitivity_rr(row$OR, row$Prevalence)
  cbind(Contaminant = row$Contaminant, result)
})

sensitivity_df <- do.call(rbind, sensitivity_results)
print(sensitivity_df)
```

6. Sensitivity Analysis Results and Interpretation

Contaminant	RR Range	PAF Range (%)	Interpretation
**Arsenic**	1.09–1.33	0.43–1.61	Very low impact on CRC burden; PAF remains under 2% even with RR variation.
**Nitrate**	1.02–1.24	0.25–3.51	Modest sensitivity; PAF increases slightly with higher RR, but remains low overall.
**THMs**	1.23–1.50	17.88–32.27	High sensitivity; PAF nearly doubles across RR range, indicating strong influence on CRC burden.
**Mercury**	0.92–1.13	–1.86–2.80	Negative PAF at lower RR suggests possible protective or null effect; high uncertainty.

**E. coli**	1.61–1.97	19.34–27.53	Consistently high PAF; strong contributor to CRC burden even under conservative RR estimates.

These results highlight that THMs and *E. coli* are the most influential and sensitive contributors to CRC burden. Mercury shows instability and high uncertainty, while Arsenic and Nitrate have limited impact across tested ranges.
